# Temporal-order judgement task suggests chronological action representations in motor experts and non-experts

**DOI:** 10.1177/1747021820936982

**Published:** 2020-07-06

**Authors:** Róisín Elaine Harrison, Martin Giesel, Constanze Hesse

**Affiliations:** School of Psychology, University of Aberdeen, Aberdeen, UK

**Keywords:** Movement perception, anticipation of future states, temporal-order judgement, athletic expertise, psychophysics

## Abstract

Motor priming studies have suggested that human movements are mentally represented in the order in which they usually occur (i.e., chronologically). In this study, we investigated whether we could find evidence for these chronological representations using a paradigm which has frequently been employed to reveal biases in the perceived temporal order of events—the temporal-order judgement task. We used scrambled and unscrambled images of early and late movement phases from an everyday action sequence (“stepping”) and an expert action sequence (“sprinting”) to examine whether participants’ mental representations of actions would bias their temporal-order judgements. In addition, we explored whether motor expertise mediated the size of temporal-order judgement biases by comparing the performances of sprinting experts with those of non-experts. For both action types, we found significant temporal-order judgement biases for all participants, indicating that there was a tendency to perceive images of human action sequences in their natural order, independent of motor expertise. Although there was no clear evidence that sprinting experts showed larger biases for sprinting action sequences than non-experts, considering sports expertise in a broader sense provided some tentative evidence for the idea that temporal-order judgement biases may be mediated by more general motor and/or perceptual familiarity with the running action rather than specific motor expertise.

## Introduction

To successfully interact with our environment, we must be able to anticipate and understand the actions of other people when planning our own actions. For example, when navigating a busy street, we must anticipate the movement directions of fellow pedestrians and adjust our own movements accordingly to avoid bumping into them. Along with our intuitions about physics and familiarity with behavioural conventions, knowledge of how human bodies usually move helps us to make predictions about potential future movements.

The anticipatory nature of movement perception has been most convincingly demonstrated by the representational momentum effect, which refers to the observation that the last remembered location of a moving stimulus is reliably displaced further along its movement path (e.g., Finke & Freyd, 1985; Freyd & Finke, 1984; Hubbard, 2005). More recently, it was found that this effect also translates to human movements (Hudson et al., 2016) and is modulated by motor expertise (Nakamoto et al., 2015). For example, basketball players showed a clear tendency to perceive the next likely state of play when provided with static images or moving videos of a basketball game (Didierjean & Marmèche, 2005; Gorman et al., 2012). Although the representational momentum effect constitutes an “error” of perception—that is, the perceived stimulus location differs from the actual stimulus location—it is assumed to function as an adaptive anticipatory mechanism that helps to extrapolate the future position of a target. The effect compensates for neural delays in the visual system, which allows us to time our actions more precisely (e.g., intercepting a thrown ball).

In contrast to simple objects in motion, such as a ball in a game, humans in general do not move along easily predictable trajectories as their movements are complex and under voluntary control. It has been hypothesised that the prediction of these complex human movements relies on internal representations that are stored in long-term memory in a structured way. Schack (2004a) hypothesised that these mental movement representations are built from several so-called Basic Action Concepts (BACs). BACs are thought to represent the most relevant action elements and body postures of a movement and are assumed to provide the basis for any kind of action anticipation. Schack and colleagues examined the categorical structure of mental representations of motor experts and non-experts in long-term memory for various sports movements, such as volleyball, golf, tennis, and gymnastics, using structural dimension analysis of motor mental representations—a technique that requires individuals to provide explicit ratings on the interrelatedness of the BACs in an action sequence (e.g., Bläsing et al., 2009; Heinen et al., 2002; Land et al., 2013; Schack, 2004b; Schack & Mechsner, 2006). The results consistently revealed that the underlying action representations were indeed spatially distinct and hierarchically ordered, and thus very similar to the real, physical actions that they represented. Furthermore, there was strong evidence that mental action representations varied with the motor expertise of individuals. More specifically, it was found that motor experts, such as athletes, possessed more detailed mental movement representations than novices for actions related to their respective field of expertise (Bläsing et al., 2009; Land et al., 2013; Schack & Mechsner, 2006). Schack and Mechsner (2006), for example, compared mental representations of the tennis serve in high-ranking tennis players, low-ranking tennis players, and novices. The results revealed that the high-ranking tennis players’ mental representations corresponded to the functional movement structure, were hierarchically organised, and were similar between individuals. Conversely, the low-ranking and novice players’ mental representations were less hierarchically organised and did not reflect the biomechanical demands of the task as precisely. These differences in mental representations between experts and non-experts suggest that motor learning leads to the development of more accurate and detailed task-specific representations, which are in turn crucial for action execution and control (Elsner & Hommel, 2001).

Importantly, if the ability to anticipate future states of a movement crucially relies on the distinct representations of action units, it seems sensible to assume that they are also organised and represented in the accurate temporal order (i.e., chronologically). Although the approach of Schack and colleagues does not allow conclusions about the representation of the temporal order of action components, there is some indirect evidence from psychophysical studies for the assumption that movement phases and components of familiar human actions are represented chronologically (e.g., Kourtzi & Shiffrar, 1999; Verfaillie & Daems, 2002). Using a priming paradigm, Kourtzi and Shiffrar (1999) presented participants with two static images (primes) of a human movement which were either linked by apparent motion or not. The first prime image depicted an early posture of a human movement whereas the second prime image depicted a later, rotated posture of the same movement. Participants were required to press a key whenever two subsequent target images matched each other. They found that participants showed priming effects for intermediate postures in both the apparent motion and static image conditions. Furthermore, there was an additional priming effect in the static image condition for target views falling outside the end of the primed motion path (i.e., for future postures). Priming effects neither occurred for target pictures preceding the presented movement nor for biomechanically impossible postures. These findings suggested that human movements are represented dynamically and in a specific spatial direction. In a later study, Verfaillie and Daems (2002) further confirmed this view by examining long-term priming of postures from movement phases. Participants were shown short animations of human-like movements in the priming phase and were later presented with static images of movement postures in the test phase. Participants were asked to determine whether the images in the test phase depicted possible or impossible body postures. They found priming effects when participants were presented with a priming animation in which the actor would have reached the test posture if the animation had lasted longer (future-posture priming) but not when they had seen an animation in which the actor would have been in the test posture if the animation had started earlier (past-posture priming). Based on these findings, they concluded that individuals anticipated future postures of observed actions and that this anticipation facilitated the subsequent perceptual identification task. Taken together, these studies suggest that human movements are represented in chronological order, which in turn seems to facilitate perceptual anticipatory processes.

As discussed above, chronologically ordered mental representations are crucial for the ability to anticipate actions. Therefore, differences in the accuracy of those representations between experts and non-experts are likely to result in differences in their anticipatory skills. Evidence for this comes from a study by Güldenpenning et al. (2012). Using a priming paradigm, they found that motor experts were more sensitive to the temporal order of expertise-related movement sequences than novices. Specifically, they presented high-jump athletes (motor experts) and non-athletes (motor novices) with prime–target pairs that depicted different body postures from a high-jump action. The high-jump action sequences were divided into different movement phases, for example, approach and flight phase, and each of these phases was further divided into four movement components (earlier to later movement components). The prime–target pairs could either show body postures selected from the same movement phase (e.g., approach and approach) or postures selected from different movement phases (e.g., approach and flight). Furthermore, the prime–target pairs were presented in either their chronological order (earlier movement as prime followed by later movement as target) or reversed order (later movement as prime followed by earlier movement as target). Participants had to indicate whether the target image depicted a posture from the approach phase or the flight phase. The results revealed a temporal-order priming effect, where participants were faster to respond to the target when prime–target pairs reflected the chronological order of the movement (e.g., approach phase prime followed by flight phase target). Importantly, although all participants showed a temporal-order priming effect for between-phase prime–target pairs (i.e., approach phase prime followed by a flight phase target), only motor experts showed a temporal-order priming effect for within-phase prime–target pairs (i.e., earlier approach phase movement followed by later approach phase movement). Güldenpenning et al. (2012) concluded that knowledge about the high-jump movement is represented in a specific (chronological) order and that more accurate mental representations may be linked to superior anticipatory skills.

In summary, the reviewed studies support the notion that movement representations are ordered chronologically as the presentation of a (static) image of a movement seems to automatically activate the visual representation of the next state of that movement, which suggests that humans have knowledge about the chronological order of familiar actions. In other words, humans expect movement sequences to appear in the order in which they commonly occur. Here, we aimed to test the existence of temporally-ordered movement representations and their influence on our perception using a novel approach. We hypothesised that our perception of temporal order might be biased when temporally ordered movement representations are activated. To investigate this, we used a temporal-order judgement task: a classical psychophysical paradigm frequently employed to examine the processing times of information in different modalities (Hendrich et al., 2012; Sternberg & Knoll, 1973) and the prioritisation of visual information (e.g., Ariga et al., 2016, for object affordances; Constable et al., 2019, for self-relevant stimuli; Rajsic et al., 2017, for valued stimuli). In our experiment, participants were presented with two images depicting different phases of a movement. The images were either presented simultaneously or separated by temporal offsets of various durations. The temporal offset separating the two images is referred to as stimulus onset asynchrony (SOA). Participants were asked to indicate which of the two images was displayed first. We hypothesised that when participants were uncertain about the presentation order due to the simultaneous presentation of the images or to short SOAs, the activation of ordered movement representations may result in a bias to prioritise movement order over the order of image presentation. In other words, we hypothesise that mental representations may act as a prior that increases the participants’ tendency to report the picture depicting the earlier movement phase to have occurred first even when it actually occurred simultaneously with, or shortly after, the picture showing a later movement phase. Note that, in theory, the temporal-order judgement task is purely perceptual as to perform this task successfully, the picture content does not have to be evaluated and motor expertise should not be required. However, as image content is difficult to ignore, it often affects performance.

As previous studies seem to suggest that there are systematic differences between the action representations of athletes and non-athletes, with athletes being more sensitive to the temporal order of movements (Güldenpenning et al., 2012) and better at anticipating future states of movements they are experts in (Aglioti et al., 2008; Gorman et al., 2012), we also tested whether and how temporal-order judgements are moderated by motor expertise. To this end, we tested a group of track and field sprinters (expert athletes) and a group of non-sprinters, with images of two different action sequences: one with which all participants should be similarly familiar (phases of a stepping movement) and one for which motor familiarity should vary between experts and non-experts (phases of a sprinting movement). We expected that track and field sprinters would show temporal-order judgement biases for both the sprinting movement (specific to their motor expertise) *and* the everyday movement (stepping). In contrast, for the non-sprinters, we predicted a similar temporal-order judgement bias as for sprinters for the everyday movement, but a significantly smaller bias for the expert sprinting movement which they should be less familiar with (i.e., they should have less accurate mental representation). We are particularly interested here in the motor experience and familiarity with sprinting movements as previous studies suggest that action capabilities affect the perception (as well as the neural processing) of actions within the field of expertise (Calvo-Merino et al., 2006; Casile & Giese, 2006). Specifically, these studies suggest that perceptual sensitivity increases for trained expert actions. The observation that motor expertise makes observers selectively sensitive to the perceptual features of those actions was coined “perceptual resonance” by Schütz-Bosbach and Prinz (2007). Thus, although most humans may be reasonably familiar with a general running movement, both perceptually and motorically, the competitive sprinters tested in our sample spent years refining their sprinting technique to optimise the distinct phases of the sprinting action depicted in our stimuli (i.e., posture during acceleration and posture during high velocity). This implies that expert sprinters possess very specific motor expertise with respect to these different phases of the sprinting movement which in turn might enhance their perceptual sensitivity to their correct chronological order.

## Methods

### Participants

Forty-five volunteers participated in the experiment. As we were interested in whether temporal-order judgements of action sequences were moderated by motor expertise, we recruited a group of participants with several years’ experience of regular training in track and field sprinting and a group of participants without any specific expertise in sprinting. Our athletic sprinting group consisted of 15 participants (9 female, mean age = 21.7 years, age range: 19–25 years) who had trained in track and field athletics for an average of 9.5 years (*SD* = 3.6 years) and have had a main training focus on sprinting for an average of 7.0 years (*SD* = 2.9 years). The mean frequency of training in the sprinting group was 5.2 sessions per week (*SD* = 0.9 sessions per week).

Thirty participants with no specific experience of track and field athletics were recruited for the non-sprinter group (23 female, mean age = 22.1 years, age range: 18–33 years). The data set of one female participant who did not understand the task instructions, performed close to chance level for all SOAs, and whose decision times were classified as outliers was excluded from analysis. Many of the participants in the non-sprinter group were also physically active (mean frequency of training = 2.4 sessions per week, *SD* = 2.0 sessions per week) and participated in a range of different sports, such as football, netball, volleyball, rugby, and mixed martial arts.

All participants reported that they had normal or corrected-to-normal vision and no neurological problems. All participants were naïve to the purpose of the experiment and provided written informed consent before the start of the experiment which lasted approximately 1 hr. The study was approved by the School of Psychology Ethics Committee at the University of Aberdeen.

### Apparatus and stimuli

The experiment was run using a Dell Precision M6500 Intel Core i5 computer (OS: Ubuntu 18.04) and programmed in MATLAB® R2018b (MathWorks, Inc.: Matick, MA, USA, 2018) using the Psychtoolbox extension (Brainard, 1997; Kleiner, 2010). Stimuli were presented on a 23.5″ LCD monitor (EIZO Foris FG2421, 52.0 × 29.5 cm, resolution: 1,920 × 1,080 pixel) with the refresh rate set to 100 Hz.

**Figure 1.**
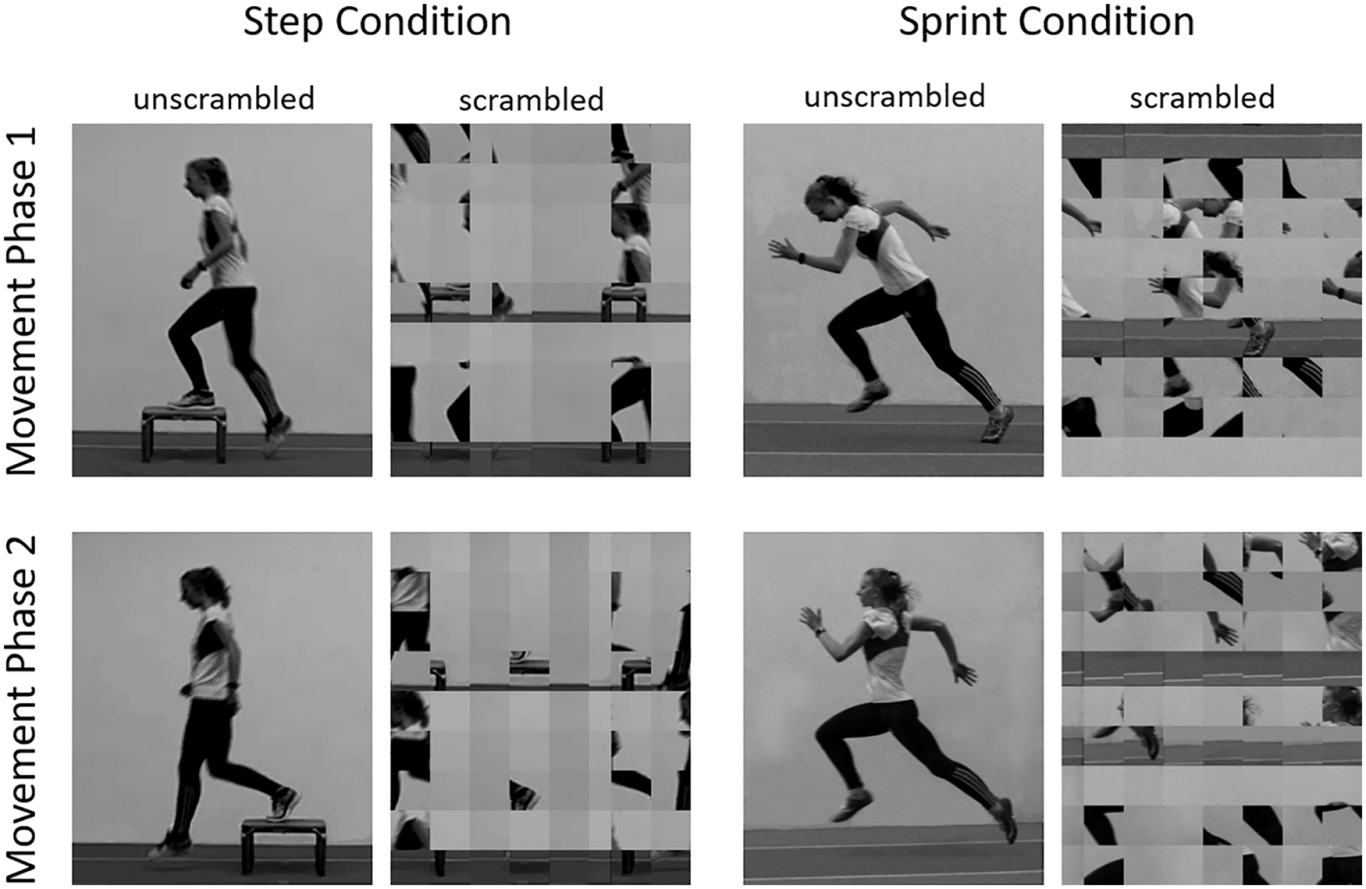
The four stimulus pairs used for the experiment. Natural movement order was characterised by Movement Phase 1 appearing before Movement Phase 2. Reversed movement order was characterised by Movement Phase 2 appearing before Movement Phase 1. In any given trial, the two images would always be from the same stimulus pair (e.g., Step Condition Phase 2 followed by Step Condition Phase 1).

The stimuli were eight grey-scale photographs (see Figure 1) which were scaled to have the same mean grey-value (0.5, mid-grey; see Figure 1). The size of each stimulus was set to 10.2 × 12.1 cm (378 × 444 pixels). Two of the images depicted a sprinting movement of a female expert, who is also the first author of this article (“sprint condition”): one image depicted the acceleration phase (Movement Phase 1) and the other depicted the maximum velocity phase (Movement Phase 2). Another two images depicted stepping movements of the same female (“step condition”): one image depicted stepping onto a box (Movement Phase 1) and the other depicted stepping off the other side of the box (Movement Phase 2). All four images depicted body postures that are representative of the respective movement phase. Stepping on and off a box was chosen as the non-expert movement as it is perceptually similar to sprinting (i.e., lifting the knee while maintaining an upright body posture) and also consists of clearly distinct phases. For both movements, the trunk is more inverted in the first phase of the action as compared with the second phase. In addition, stepping and sprinting are both cyclical actions but consist of higher order phases allowing a categorical distinction between the different sequences.

The remaining four images were scrambled versions of the image pairs in the sprint condition (“sprint-scrambled condition”) and the step condition (“step-scrambled condition”), respectively. The scrambled images were created from the original images by first dividing each of them in as many blocks of 50 × 50 pixel as possible and then randomly repositioning these blocks as well as the remainder of smaller blocks. The rationale for this method of creating the scrambled images was to effectively obscure the type of movement and movement phase while keeping the low-level features of the scrambled images as similar to the original images as possible (e.g., perceived contrast). We piloted pixel-wise scrambling, but the images appeared largely identical and homogenously grey (i.e., white noise) with this method. Consequently, we decided to use larger blocks as the images still contained recognisable features of the moving person and the surround—thereby making the scrambled images similar in salience to the original images. Note that in each trial of the experiment, we always presented image pairs belonging to the same condition (i.e., Movement Phases 1 and 2 from either the sprint, step, sprint-scrambled, or step-scrambled condition). The two stimuli were presented on a mid-grey background and horizontally centred on the screen. The vertical distance between the stimuli from the centre of the screen was ±50 pixels (1.35 cm).

### Procedure

Participants sat at a table in a darkened room at a viewing distance of 75 cm from the monitor. A height-adjustable chin rest was used to maintain a constant viewing distance throughout the experiment. A button box with two buttons arranged in vertical order was placed on the table in front of the participants. They were instructed to hold the box with both hands and place the index fingers (or thumbs) of each hand on the upper and lower buttons, respectively. To start a trial, participants pressed both buttons simultaneously. A black fixation cross (50 × 50 pixel) appeared in the centre of the screen and remained there until the end of the trial. Subsequently, one of the four stimulus pairs appeared on the screen. The two images could either be presented simultaneously (SOA: 0 ms) or with a short temporal offset between them (SOA: 30, 50, or 100 ms). Both images remained visible on the screen together for a duration of 500 ms. After this interval, the stimuli were replaced by a response screen, and participants were required to indicate which image they thought had appeared first on the screen by pressing the corresponding button on the button box (i.e., if they thought that the image presented above the fixation cross appeared first, they pressed the top button on their button box and vice versa). As the picture content was irrelevant to the task, participants were not made aware of the presentation of different movement phases and types.

The presentation sequence of SOA, image type (i.e., “sprint,” “sprint-scrambled,” “step,” “step-scrambled”), and presentation location of the first image (i.e., below or above the fixation cross) was randomised for each participant. Seven SOAs were employed in this experiment: –100, –50, –30, 0, +30, +50, and +100 ms. Positive SOAs indicate that the images were presented in their natural movement order (i.e., the image depicting Movement Phase 1 was followed by the presentation of the image depicting Movement Phase 2), whereas negative SOAs indicate that the images were presented in reversed movement order (i.e., the image depicting Movement Phase 2 was presented first). The image that was presented first appeared equally often in the top half of the screen and the bottom half of the screen. All of these manipulations generated a total of 56 different combinations: 7 SOAs × 2 locations (top or bottom) × 4 image types. Each of these combinations was presented 20 times resulting in 1,120 experimental trials in total. After every 50 trials, a screen would appear to encourage participants to take a short break.

Prior to the start of the main experiment, participants completed a short practice session to become accustomed to the task. The practice trials followed the same procedure as the experimental trials but used different stimuli (images of mugs), and a constant SOA of 100 ms between the appearance of the first and second image. In addition, participants received auditory feedback about their performance during practice (beeps with a duration of 250 ms; high-pitched for correct responses [1,000 Hz] and low pitched [500 Hz] for incorrect responses). Participants indicated verbally to the experimenter when they felt familiar with the task and wished to begin the experimental session during which no performance feedback was given.

### Data processing and analysis

To analyse the data, we determined the point of subjective simultaneity (PSS) for each participant and image condition. The PSS indicates the SOA at which a participant would have perceived the images as being presented in their natural order in 50% of the trials. We first computed the proportion of trials in which a participant perceived the images as being presented in their natural movement order (i.e., Movement Phase 1 followed by Movement Phase 2) separately for each participant, image type, and SOA. The proportions of perceived natural movement order were then used to fit psychometric functions (cumulative normal functions) using the Palamedes toolbox (Prins & Kingdom, 2018). Thresholds and slopes were free parameters in the fit while the guess rate was fixed at 0 and lapse rate at 0.01. A negative PSS indicates a tendency to perceive images as appearing in their natural movement order despite being presented simultaneously or in reversed order, whereas a positive PSS indicates a tendency to perceive images as appearing in reversed movement order despite being presented in their natural order. For example, a PSS of −5 ms would indicate that participants showed a temporal-order judgement bias in the expected direction and would be predicted to perform at chance level if Movement Phase 2 was presented 5 ms before Movement Phase 1 (i.e., SOA of −5 ms).

In addition, we analysed the time it took participants to provide their answer (i.e., decision time). The decision time reflects the time between the appearance of the response screen after the presentation of the images and the moment participants provided their button-press response.

The PSS-data were initially analysed using a 2 × 2 × 2 mixed analysis of variance (ANOVA) with the within-subject factors *movement type* (sprint vs. step) and *scrambling* (scrambled vs. unscrambled) and the between-subject factor *sprinting expertise* (sprinter vs. non-sprinter). All post hoc tests were conducted one-sided (as all our hypotheses predict a clear direction of the order of effects) and were Bonferroni corrected for multiple comparisons, if applicable. All values are presented as means ± 1 SEMs (standard errors of the mean). A significance level of α = .05 was used for all statistical analyses.

## Results

### PSS

**Figure 2.**
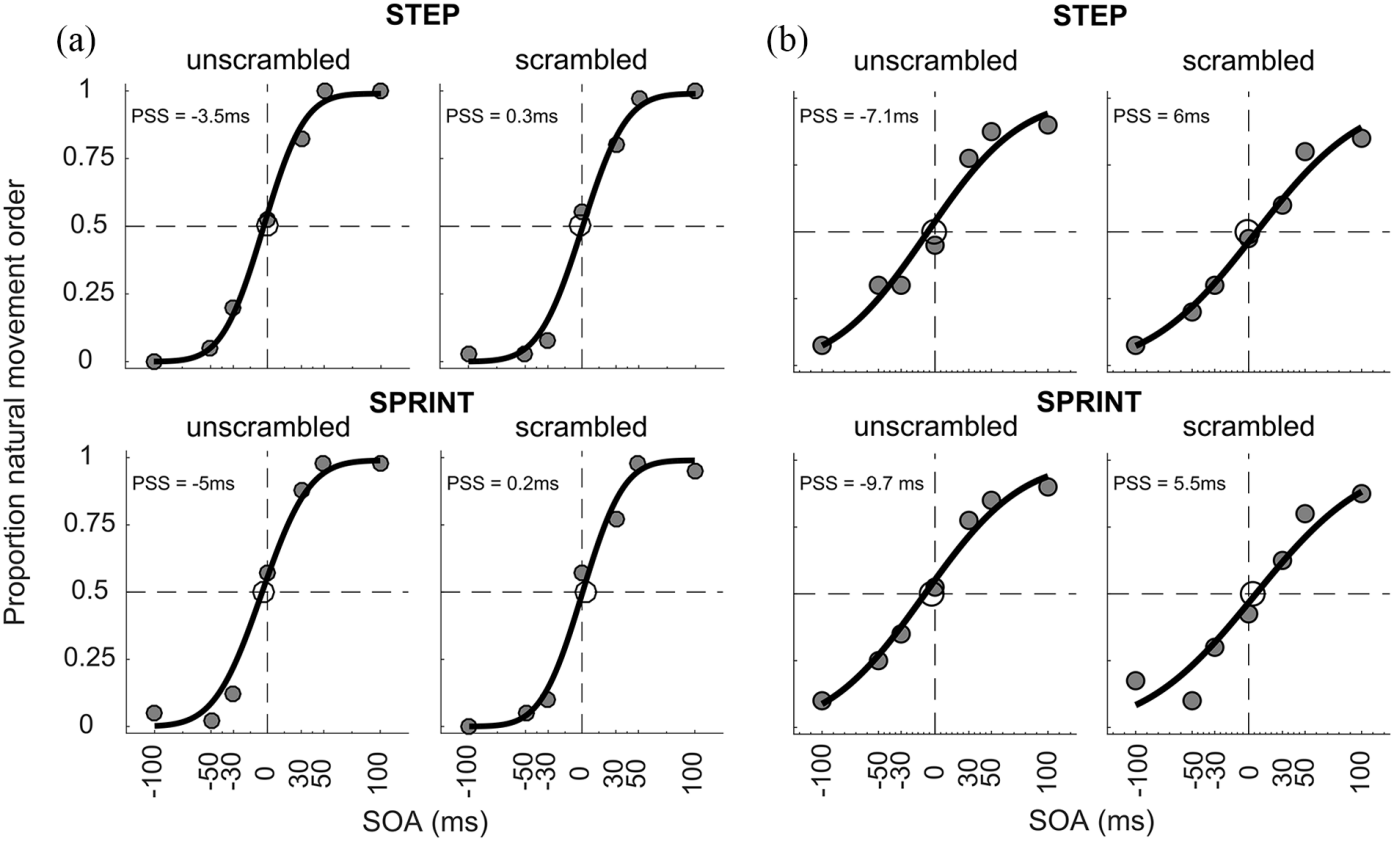
Psychometric functions for two different participants (a and b) who showed a temporal-order judgement bias in both the step and sprint conditions. The upper row shows the results for the STEP condition, and the lower row shows results for the SPRINT condition. Grey data points indicate the proportion of responses where the image showing Movement Phase 1 was judged as being presented before the image depicting Movement Phase 2. A negative PSS indicates a bias towards perceiving the images in their chronological order.

**Figure 3.**
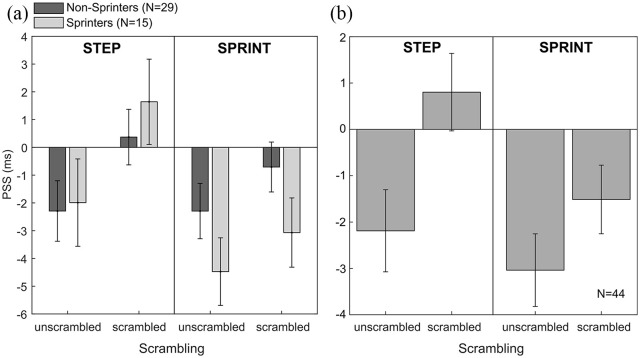
(a) Points of subjective simultaneity (PSS) for all four image type conditions in the two expert groups. Negative values indicate a temporal-order judgement bias such that an image representing the first movement phase is perceived as being presented first even though it occurred second. (b) PSS averaged across both groups. Error bars represent ±1 SEM between participants.

Figure 2 shows the data and fitted psychometric functions of two representative participants who showed a temporal-order judgement bias for both the step and sprint conditions. The 2 × 2 × 2 mixed ANOVA on the PSS values revealed a main effect of *movement type, F*(1, 42) = 4.82, *p* = .034, $\eta_{p}^{2}$ = .10, and a main effect of *scrambling, F*(1, 42) = 9.76, *p* = .003, $\eta_{p}^{2}$ = .19. The main effect of *movement type* indicates that across both scrambling conditions, the temporal-order judgement bias was slightly larger for the sprinting images (–2.6 ± 0.57 ms) than for the stepping images (–0.57 ± 0.77 ms). More importantly, the main effect of *scrambling* indicates that the PSS reflected, as expected, a larger temporal-order judgement bias for unscrambled images (–2.8 ± 0.68 ms) than for scrambled images (–0.4 ± 0.54 ms). There was no main effect of *sprinting expertise* (*p* = .45) and no significant interaction effects between any of the factors (all *p* > .11). Thus, contrary to our hypothesis that sprinting experts might show a selectively larger temporal-order judgement bias in the sprint condition than non-sprinters, there was no three-way interaction between the variables. Descriptively, it appears that sprinting experts showed larger temporal-order judgement biases for both the sprint and the sprint-scrambled conditions (Figure 3a, see “Discussion” for further information).

Importantly, however, the finding that there was a main effect of scrambling seems to suggest that our sample, as a whole, showed a temporal-order judgement bias and thus a tendency to perceive earlier movement phases as being presented first for both of the action sequences. To test whether a temporal-order judgement bias occurred reliably across the entire sample, we averaged the data across both groups: sprinters and non-sprinters (see Figure 3b).

To determine the existence of a temporal-order judgement bias (which would be reflected in negative PSS values), one-sample *t* tests comparing the PSS against zero were conducted for each of the four image types (note that the ANOVA only tests for differences between conditions but *does not* provide information on whether values are larger or smaller than zero and thus, does not determine whether a temporal-order judgement bias exists in the expected direction).

For the stepping condition, we found a significant temporal-order judgement bias for the unscrambled images, *t*(43) = –2.47, *p* = .034, *d* = 0.37 (Bonferroni corrected), but not for the scrambled versions, *t*(43) = –0.96, *p* = .684, *d* = 0.14. Similarly, for the sprint condition, PSS were also significantly smaller than zero in the unscrambled condition, *t*(43) = –3.88, *p* < .001, *d* = 0.58, but not in the scrambled condition, *t*(43) = –2.04, *p* = .094, *d* = 0.31. Overall, these findings seem to indicate that participants show statistically significant temporal-order judgement biases for both action sequences tested, independent of their motor expertise.

**Figure 4.**
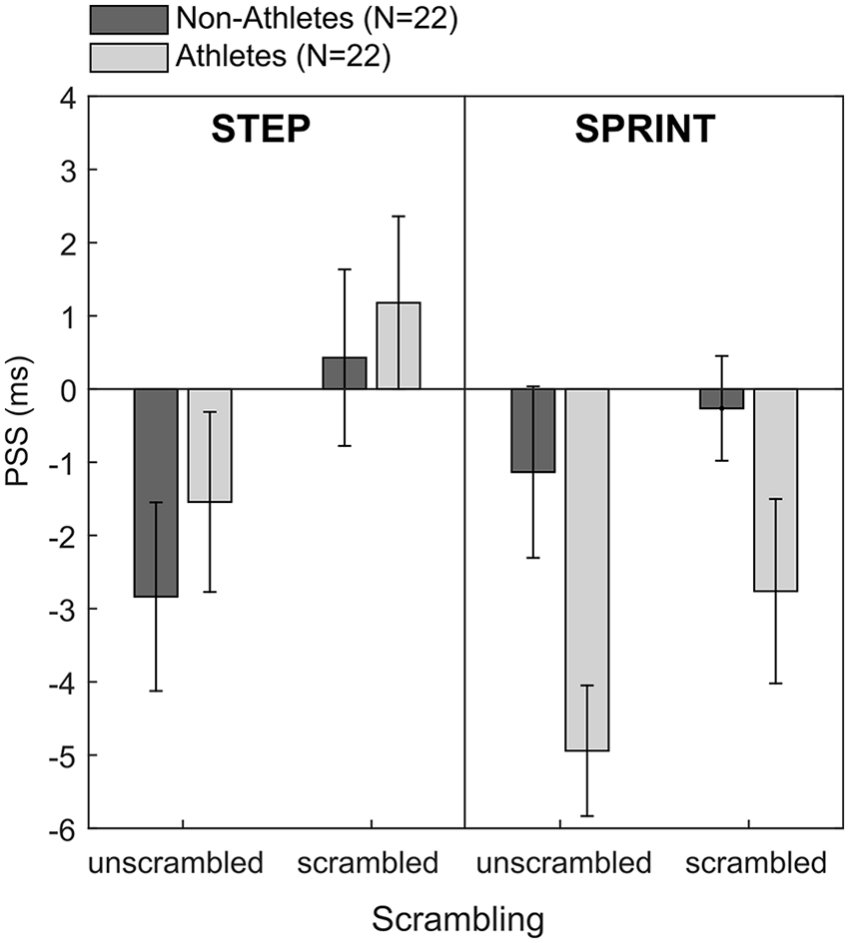
Points of subjective simultaneity (PSS) for all four image type conditions and the recoded expert groups. Error bars represent ±1 SEM.

As a number of participants in the non-sprinting sample still had considerable experience in other sports, we wondered whether sports expertise defined more broadly may moderate the temporal-order judgement bias for the different movement types. To explore this, we recoded our sample according to their general sports expertise. Every participant who trained consistently for a certain sport at least four times per week was coded as an “athlete.” Most of these participants participated in team-sports that involved sprinting and running such as football, rugby, volleyball, and netball. There were, however, three participants who performed sports at a competitive level but whose primary sport did not involve a significant element of running (i.e., a dancer, a mixed martial arts and ballet performer, and a competitive horse rider). We decided to keep these three participants in the athlete sample because we deemed it likely that these participants also incorporated running into their general health, training, and exercise regime (which we did not assess and cannot determine retrospectively). In addition, their primary sports are extremely posture-oriented which may increase these participants’ perceptual sensitivity to body postures in general. Therefore, we felt that these three participants fitted in better with the athlete sample than the non-athlete sample. This resulted in a more even split of our sample with 22 participants assigned to the athlete and non-athlete groups, respectively. For this recoded sample, we re-computed the 2 × 2 × 2 mixed ANOVA which confirmed the main effect of scrambling, *F*(1, 42) = 10.28, *p* = .003, $\eta_{p}^{2}$ = .20. In addition, the analysis showed an interaction effect between *movement type* and *sports expertise, F*(1, 42) = 5.85, *p* = .02, $\eta_{p}^{2}$ = .12. Figure 4 shows that this interaction effect seems to be mainly driven by the fact that athletes showed overall larger temporal-order judgement biases in the expected direction in the sprint condition than non-athletes. Surprisingly, this seems to be true for both unscrambled and scrambled sprinting images, suggesting that athletes might still have been able to detect some features in the scrambled pictures that indicated movement order (see “Discussion” for more information).

The finding that temporal-order judgement biases were generally larger for athletes than non-athletes for images in the sprint condition may thus provide some tentative evidence for the notion that the size of the temporal-order judgement bias may be mediated by more general motor and/or perceptual familiarity with the running movement. The main effects of *movement type* (*p* = .07) and *sports expertise* (*p* = .25) were not significant. There were no other significant interaction effects (all *p* > .31).

### Decision time

**Figure 5.**
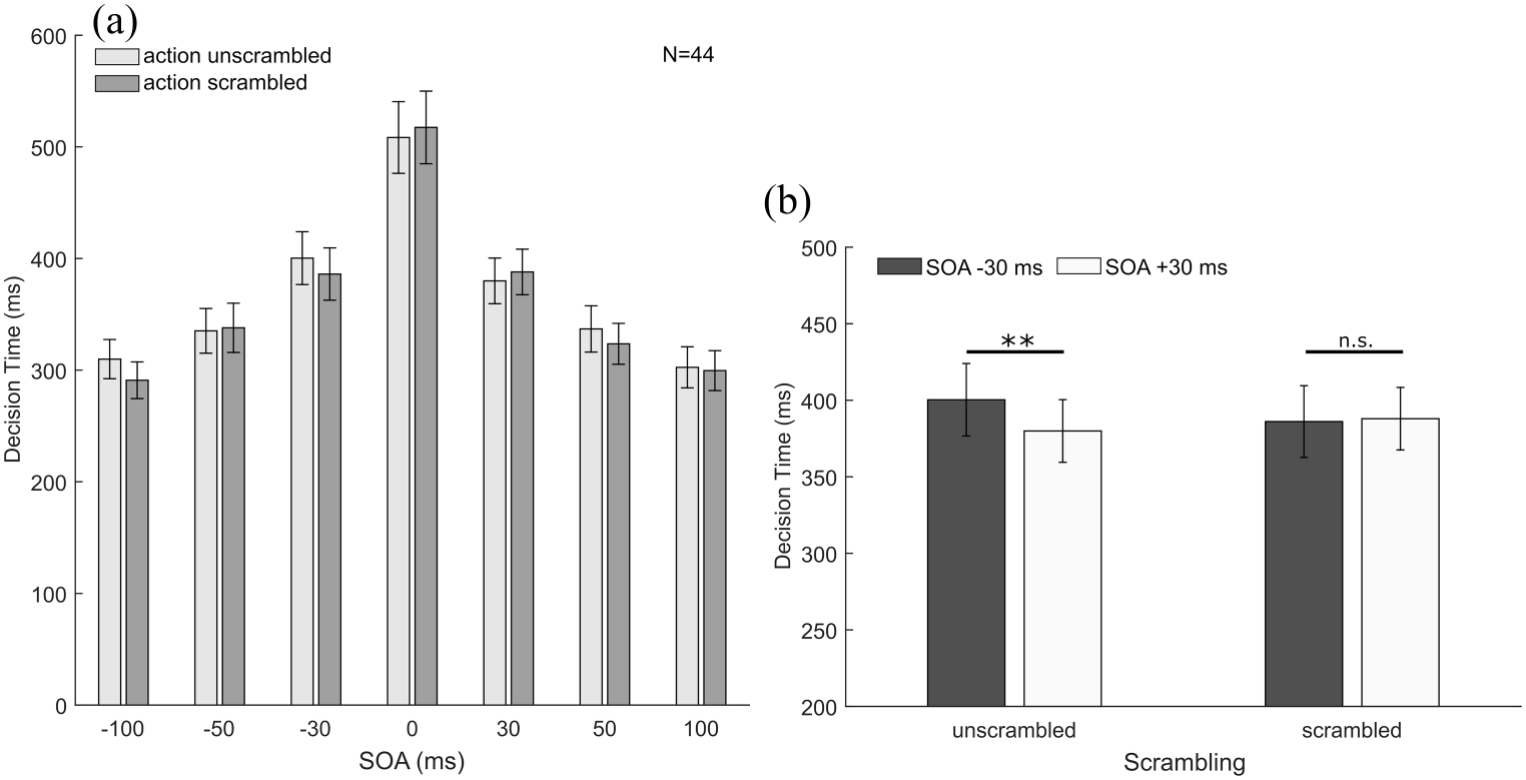
(a) Decision time as a function of scrambling and SOA. Participants’ responses became faster the longer the SOA between the presentations of the two images. (b) Decision time for the unscrambled and scrambled images at the shortest SOA of 30 ms. Negative SOAs indicate that images were presented in the reverse movement order (i.e., second movement phase presented first) and positive SOAs indicate that images were presented in their natural order (i.e., first movement phase presented first). For unscrambled actions, participants showed quicker responses when images were presented in their natural order. Error bars represent ±1 SEM between participants.

As a pre-analysis of the data revealed that the two different types of movements displayed in the images (i.e., step vs. sprint) had no effect on the decision times, data were averaged across this factor. Moreover, as there was no main effect of the between-subject factor “expertise” or interactions between “expertise” and any of the other factors (neither when defined as the original sprinter sample nor when defined as the recoded athlete sample), the final analysis was conducted across the whole sample. The data are shown as a function of *SOA* and *scrambling* in Figure 5a. As can be seen in this figure, decision times were longest when both images were presented simultaneously and decreased considerably for longer SOAs. Note that participants were given no instructions about the speed with which they had to provide their answers and that answers were only recorded after the response screen had been displayed (see “Methods” section). Nevertheless, participants’ decision times decreased considerably when SOAs increased (and hence for easier trials). We were particularly interested in whether presenting movements in their natural order (i.e., with no conflict between the order of presentation and the order of the action sequence depicted in the images) may lead to faster responses (facilitation effect) while presenting images in an order depicting reverse action sequences (i.e., with a conflict between order of presentation and order of image content) may lead to prolonged decision times (interference effect). To determine this, we conducted a 2 (*scrambling*) × 2 (*presentation order*: natural vs. reversed) × 3 (*SOA duration*: 30, 50, and 100 ms) repeated-measures ANOVA. Note that the SOA = 0 ms condition was omitted from this analysis as in this condition both images were presented simultaneously. As expected, this analysis revealed a strong main effect of *SOA duration, F*(2, 86) = 56.05, *p* < .001, $\eta_{p}^{2}$ = .57, with decision times decreasing for longer SOAs. Importantly, there was also a significant three-way interaction between all factors, *F*(2, 86) = 6.12, *p* = .003, $\eta_{p}^{2}$ = .13. Due to this three-way interaction effect, the main effects of *scrambling* (*p* = .04) and *presentation order* (*p* = .047) cannot be meaningfully interpreted. All other interaction effects were not statistically significant (all *p* > .20). The three-way interaction suggests that SOA duration and presentation order had differential effects for scrambled and unscrambled images. To better understand this three-way interaction, we conducted for each SOA (i.e., 30, 50, and 100 ms) separate repeated-measures ANOVA with the factors *scrambling* and *presentation order*. For the 30 ms SOA, this analysis revealed a significant interaction effect, *F*(1, 43) = 6.00, *p* = .019, $\eta_{p}^{2}$ = .12. Paired-samples *t* tests confirmed that participants were about 20 ± 6 ms faster to provide their responses when unscrambled images were presented in their natural order (positive SOA) than when they were shown in reversed order, *t*(43) = 3.27, *p* = .004, *d* = 0.49. In contrast, decision times were unaffected by the order of presentation for scrambled images, *t*(43) = 0.26, *p* = .80, *d* = 0.04 (see Figure 5b). Thus, for the shortest SOA condition (±30 ms) in which participants should be most uncertain about the order in which the images had been presented, decision times increased if there was a conflict between the order of presentation and the order of the action sequences depicted in the presented images. The same analysis for the 50 ms SOA condition and the 100 ms SOA condition revealed no significant interaction effects between scrambling and presentation order (all *p* > .06). For the 100 ms SOA condition, we observed a significant effect of scrambling, *F*(1, 43) = 9.00, *p* = .004, $\eta_{p}^{2}$ = .17, that indicated that participants were slightly slower to respond when unscrambled images were presented (306 ± 18 ms) as compared with scrambled ones (295 ± 17 ms).

## Discussion

The aim of this study was to determine whether individuals are biased towards perceiving images of human movement phases as appearing in their natural movement order, and, if this is the case, whether these temporal-order judgements are influenced by motor expertise. We investigated these questions by presenting images of stepping and sprinting action sequences to sprinters and non-sprinters in a temporal-order judgement task. We predicted that participants would show a temporal-order judgement bias, meaning that at short SOAs, they should show an increased tendency to rate pictures depicting earlier movement phases to have been presented before pictures showing later movement phases, even if they were actually presented simultaneously or second. We further hypothesised that the size of bias would be moderated by motor familiarity with the movement. Specifically, we predicted that the sprinters would show temporal-order judgement biases for both the sprinting action (specific to their motor expertise) *and* the everyday action (stepping), whereas the non-sprinters were expected to show a significantly smaller bias for the sprinting action, compared with the stepping action and also compared with the sprinters. Our results suggest that, regardless of motor expertise, participants show a significant bias towards perceiving images of movement phases in the order in which they naturally occur. However, we found no significant differences in the size of the temporal-order judgement biases of sprinters and non-sprinters for expertise-related actions.

The main novel finding of our study is that participants exhibited temporal-order judgement biases for images of action sequences. This finding provides the first direct experimental evidence for the idea that mental representations of movements are chronological in nature. In the temporal-order judgement task, we created a conflict between the expected order in which movements occur and the order in which these movements were presented. When there is high perceptual uncertainty about presentation order, this conflict may result in mental movement representations overriding perceptual signals and therefore guiding the judgement of temporal order. In other words, participants’ mental representations of movements were strong enough to change temporal-order perception when images of movement phases were presented (in contrast to the scrambled pictures), despite these movements being task-irrelevant. Although the magnitude of the temporal-order judgement biases found in the current study might seem small, they are of comparable size with those observed in previous studies using the temporal-order judgement task to measure the prioritisation of visual information (e.g., Ariga et al., 2016; Constable et al., 2019).

Our findings add to the motor priming literature that revealed temporal-order priming effects for human movements and support their suggestion that human movements are represented in their natural temporal order (e.g., Güldenpenning et al., 2012; Kourtzi & Shiffrar, 1999; Verfaillie & Daems, 2002). While priming studies demonstrate that humans anticipate future movement phases when presented with static images of action sequences, the temporal-order judgement task measures directly how perception of temporal order is biased by our implicit expectations. The temporal-order judgement bias is likely to be the result of adaptive processes that consolidate chronological movement representations. Although this results in an erroneous perception in the artificial temporal-order judgement task (creating a conflict between perceptual order and naturally occurring movement order which is unlikely to be observed in real life), it is advantageous in real life as these representations are thought to aid the anticipation of movements (Güldenpenning et al., 2012; Schack et al., 2016).

Regarding decision times, we found that participants’ decision times were longest for simultaneous presentations (SOA = 0 ms) and shortened with increasing SOAs both for scrambled and unscrambled images. More interestingly, the decision time data also provided further evidence that natural movement order affected temporal-order judgements. At the two shortest SOAs (±30 ms), participants tended to respond faster when unscrambled images were presented such that there was no conflict between the presentation order and the natural order of the depicted movement (i.e., for SOA = + 30 ms). In contrast, their decision times increased when the presentation order was the inverse of the natural movement order (i.e., for SOA = –30 ms). Note that this effect only occurred for the ±30 ms SOAs. For longer SOAs, we observed no facilitation or interference effects of natural movement order on decision times for temporal-order judgements. The asymmetry in decision times between the ±30 ms SOAs for unscrambled pictures (longer for −30 ms and shorter for + 30 ms) can be seen as a consequence of the negative PSS (resulting from a temporal-order judgement bias towards natural movement order). For unbiased participants, whose judgements are just based on presentation order and not influenced by natural movement order, we would expect no difference in task difficulty for positive and negative SOAs with the same duration. The task should be most difficult for the 0 ms SOA, and then difficulty should decrease symmetrically for negative and positive SOAs. Accordingly, we would expect the longest decision times for the 0 ms SOA and symmetrically decreasing decision times for longer SOAs (i.e., similar duration for positive and negative SOAs). For a biased participant, however, the task should be most difficult at the (non-zero) PSS. The negative PSS that we found is considerably closer to the 0 ms SOA than to the next longer SOA (–30 ms), so we would still expect the task to be most difficult at the 0 ms SOA. However, the −30 ms SOA is closer to the PSS than the +30 ms SOA and therefore should have a higher task difficulty. Our analysis of the decision time data for the ±30 ms SOAs shows exactly the behaviour expected for biased participants for the unscrambled images, and performance for the scrambled images is in line with the behaviour expected for unbiased participants. With further increasing SOA duration, the presentation order begins to dominate perception, and the decision times show no longer a statistically significant effect of the temporal-order judgement bias.

The second question of our study concerned the moderating effect of motor expertise. Specifically, we aimed to test whether the size of the temporal-order judgement bias would be larger for sprinting athletes who have high motor familiarity with the movement and the different body postures due to years of training. Previous studies have shown that motor training and motor familiarity affect the perception of trained movements (e.g., Casile & Giese, 2006) as well as the neural processing of them (Calvo-Merino et al., 2006) even when there are no differences in the perceptual familiarity between motor experts and non-experts with those movements. For our study, this means that even if experts and non-experts are both perceptually similarly familiar with the movement, that is, they all have seen and observed a large number of human sprinting and running movements in their lifetime, their sensitivity to temporal order may still differ due to differences in their motor familiarity and expertise with these movements. More generally, the perceptual processing and sensitivity to actions is thought to be moderated by the motor ability to produce them (Schütz-Bosbach & Prinz, 2007). Yet, our data provided no evidence for this assumption. We found that PSS values did not statistically differ between sprinters and non-sprinters for both movement type conditions (i.e., sprinting and stepping images).

Although track and field sprinting at a competitive level involves years of technical training to optimise the different movement phases (i.e., acceleration and high velocity), the general body postures and their temporal order are very similar for all forms of running. That is, to accelerate, the body must be inclined to pick up some speed; followed by a phase of “upright running” at a constant high speed. Thus, this more general motor and/or perceptual familiarity with the running movement may be sufficient to elicit a temporal-order judgement bias. We found some tentative evidence for this idea when we recoded our sample to include participants who either very regularly performed sports that involved running or sprinting as part of a team game, or sports that were very posture-oriented (such as dancing or mixed martial arts). For this recoded sample, we found larger temporal-order judgement biases for athletes as compared with non-athletes for the sprinting images but independent of scrambling (see Figure 4). Still, as this recoding was done post hoc, these findings should be interpreted with caution. The fact that athletes tended to show larger temporal-order judgement biases for sprinting movements—independent of scrambling—may suggest that they were still able to identify certain features in the scrambled images that indicate movement phases and thus may have been able to perceive, to some extent, temporal order in those pictures. Note, that we used relatively large blocks for scrambling (i.e., 50 × 50 pixels) and presented the same images across all trials and conditions (instead of scrambling images on a trial-by-trial basis). Even though this may have confounded our current data, the finding is in itself interesting as it may indicate that there is a difference between athletes’ and non-athletes’ perception of human movement and that athletes may require a higher level of scrambling than non-athletes to no longer recognise action sequences and their temporal order, in particular in images with high motor and/or perceptual familiarity. Future research could address this question by presenting images of human movement with varying degrees of scrambling and investigating whether the perceptual threshold for discriminating human movement differs between motor experts and non-experts.

As general running/athletic expertise rather than motor expertise specific to track sprinting seemed to moderate the extent to which perception was influenced by temporal-order information inherent in the sprinting images, the question arises if perhaps expertise-modulated performance differences only begin to emerge when the task is sufficiently difficult. For example, Güldenpenning et al. (2012) found that both motor experts and non-experts exhibited a temporal-order priming effect for between-movement-phase stimulus pairs (e.g., approach vs. flight) depicting postures from a high-jump movement, but only high-jump athletes demonstrated a temporal-order priming effect for within-movement-phase stimulus pairs (e.g., early vs. late approach). Thus, it was only with the use of within-movement-phase stimulus pairs that the performance of athletes and non-athletes began to diverge. As the present study used between-movement-phase stimulus pairs (i.e., acceleration and maximum velocity), it is possible that by using within-movement-phase stimulus pairs (e.g., earlier acceleration posture and later acceleration posture) differences in performance may arise between sprinting experts and non-experts. Related to this question, one reviewer of this article raised the question of whether or not temporal order is easily identifiable for non-experts in both our sprinting and stepping stimuli. To address this issue, we presented our stimulus pairs to a large number of observers (*N* = 63) using an online questionnaire. The scrambled image pairs were presented first (with sprinting and stepping counterbalanced) followed by the unscrambled pairs (again both movement types were counterbalanced). Observers were asked to judge which image would come first and which second in a movement sequence. We found that all observers correctly identified the temporal order for the unscrambled sprinting pictures and all, but one, for the unscrambled stepping pictures. As for the scrambled pictures, the correct order was identified by 49.2% of the sample for stepping and 57.1% for the sprinting pictures, suggesting that scrambling successfully obscured the temporal order of the movements. This further highlights the necessity of future studies to test movements that are less common and more specific to participants’ expertise (e.g., pole vault, pirouette) to examine if there are reliable effects of expertise on temporal-order judgement biases in these instances.

In conclusion, our study provides novel evidence that depicted movement order can influence temporal-order judgements. All participants showed a bias towards perceiving sprinting and stepping movements in their natural order. The question of whether and how this effect is moderated by expertise could not be answered conclusively. In sum, these findings support the notion that the mental representations of actions are chronological.

## References

[bibr1-1747021820936982] Aglioti S. M. Cesari P. Romani M. Urgesi C. (2008). Action anticipation and motor resonance in elite basketball players. Nature Neuroscience, 11(9), 1109–1116. 19160510 10.1038/nn.2182

[bibr2-1747021820936982] Ariga A. Yamada Y. Yamani Y. (2016). Early visual perception potentiated by object affordances: Evidence from a temporal order judgment task. i-Perception, 7(5), 2041669516666550. 27698991 10.1177/2041669516666550 PMC5030742

[bibr3-1747021820936982] Bläsing B. Tenenbaum G. Schack T. (2009). The cognitive structure of movements in classical dance. Psychology of Sport and Exercise, 10(3), 350–360.

[bibr4-1747021820936982] Brainard D. H. (1997). The psychophysics toolbox. Spatial Vision, 10, 433–436. 9176952

[bibr5-1747021820936982] Calvo-Merino B. Grèzes J. Glaser D. E. Passingham R. E. Haggard P. (2006). Seeing or doing? Influence of visual and motor familiarity in action observation. Current Biology, 16(19), 1905–1910. 17027486 10.1016/j.cub.2006.07.065

[bibr6-1747021820936982] Casile A. Giese M. A. (2006). Nonvisual motor training influences biological motion perception. Current Biology, 16(1), 69–74. 16401424 10.1016/j.cub.2005.10.071

[bibr7-1747021820936982] Constable M. D. Welsh T. N. Huffman G. Pratt J. (2019). I before U: Temporal order judgements reveal bias for self-owned objects. Quarterly Journal of Experimental Psychology, 72(3), 589–598. 10.1177/1747021818762010 29431023

[bibr8-1747021820936982] Didierjean A. Marmèche E. (2005). Anticipatory representation of visual basketball scenes by novice and expert players. Visual Cognition, 12(2), 265–283.

[bibr9-1747021820936982] Elsner B. Hommel B. (2001). Effect anticipation and action control. Journal of Experimental Psychology: Human Perception and Performance, 27(1), 229–240. 11248937 10.1037//0096-1523.27.1.229

[bibr10-1747021820936982] Finke R. A. Freyd J. J. (1985). Transformations of visual memory induced by implied motions of pattern elements. Journal of Experimental Psychology: Learning, Memory, and Cognition, 11(4), 780–794. 10.1037//0278-7393.11.1-4.780 2932525

[bibr11-1747021820936982] Freyd J. J. Finke R. A. (1984). Representational momentum. Journal of Experimental Psychology: Learning, Memory, and Cognition, 10(1), 126–132.

[bibr12-1747021820936982] Gorman A. D. Abernethy B. Farrow D. (2012). Classical pattern recall tests and the prospective nature of expert performance. The Quarterly Journal of Experimental Psychology, 65(6), 1151–1160. 22414197 10.1080/17470218.2011.644306

[bibr13-1747021820936982] Güldenpenning I. Kunde W. Weigelt M. Schack T. (2012). Priming of future states in complex motor skills. Experimental Psychology, 59(5), 286–294. 22617314 10.1027/1618-3169/a000156

[bibr14-1747021820936982] Heinen T. Schwaiger J. Schack T. (2002, 7 24–28). Optimising gymnastics training with cognitive methods [Paper presentation]. Proceedings of 7th Annual Congress of the European College of Sport Science, Athens, Greece.

[bibr15-1747021820936982] Hendrich E. Strobach T. Buss M. Mueller H. J. Schubert T. (2012). Temporal-order judgment of visual and auditory stimuli: Modulations in situations with and without stimulus discrimination. Frontiers in Integrative Neuroscience, 6, 63. 22936902 10.3389/fnint.2012.00063 PMC3427541

[bibr16-1747021820936982] Hubbard T. L. (2005). Representational momentum and related displacements in spatial memory: A review of the findings. Psychonomic Bulletin & Review, 12(5), 822–851. 16524000 10.3758/bf03196775

[bibr17-1747021820936982] Hudson M. Nicholson T. Simpson W. A. Ellis R. Bach P. (2016). One step ahead: The perceived kinematics of others’ actions are biased toward expected goals. Journal of Experimental Psychology: General, 145(1), 1–7. 26595838 10.1037/xge0000126 PMC4694084

[bibr18-1747021820936982] Kleiner M. (2010, 8 22–26). Visual stimulus timing precision in Psychtoolbox-3: Tests, pitfalls and solutions [Paper presentation]. 33rd European Conference on Visual Perception (ECVP 2010), Lausanne, Switzerland.

[bibr19-1747021820936982] Kourtzi Z. Shiffrar M. (1999). Dynamic representations of human body movement. Perception, 28(1), 49–62. 10627852 10.1068/p2870

[bibr20-1747021820936982] Land W. Volchenkov D. Bläsing B. E. Schack T. (2013). From action representation to action execution: Exploring the links between cognitive and biomechanical levels of motor control. Frontiers in Computational Neuroscience, 7, 127. 24065915 10.3389/fncom.2013.00127 PMC3776155

[bibr21-1747021820936982] Nakamoto H. Mori S. Ikudome S. Unenaka S. Imanaka K. (2015). Effects of sport expertise on representational momentum during timing control. Attention, Perception, & Psychophysics, 77(3), 961–971. 10.3758/s13414-014-0818-9 25537739

[bibr22-1747021820936982] Prins N. Kingdom F. A. (2018). Applying the model-comparison approach to test specific research hypotheses in psychophysical research using the Palamedes Toolbox. Frontiers in Psychology, 9, 1250. 30083122 10.3389/fpsyg.2018.01250 PMC6064978

[bibr23-1747021820936982] Rajsic J. Perera H. Pratt J. (2017). Learned value and object perception: Accelerated perception or biased decisions? Attention, Perception, & Psychophysics, 79(2), 603–613. 10.3758/s13414-016-1242-0 27896709

[bibr24-1747021820936982] Schack T. (2004a). The cognitive architecture of complex movement. International Journal of Sport and Exercise Psychology, 2(4), 403–438.

[bibr25-1747021820936982] Schack T. (2004b). Knowledge and performance in action. Journal of Knowledge Management, 8(4), 38–53.

[bibr26-1747021820936982] Schack T. Mechsner F. (2006). Representation of motor skills in human long-term memory. Neuroscience Letters, 391(3), 77–81. 16266782 10.1016/j.neulet.2005.10.009

[bibr27-1747021820936982] Schack T. Schütz C. Krause A. F. Seegelke C. (2016). Representation and anticipation in motor action. In Nadin M. (Ed.), Anticipation across disciplines (pp. 203–215). Springer.

[bibr28-1747021820936982] Schütz-Bosbach S. Prinz W. (2007). Perceptual resonance: Action-induced modulation of perception. Trends in Cognitive Sciences, 11(8), 349–355. 17629544 10.1016/j.tics.2007.06.005

[bibr29-1747021820936982] Sternberg S. Knoll R. L. (1973). The perception of temporal order: Fundamental issues and a general model. In Kornnblum S. (Ed.), Attention and performance IV (pp. 629–685). Academic Press.

[bibr30-1747021820936982] Verfaillie K. Daems A. (2002). Representing and anticipating human actions in vision. Visual Cognition, 9(1–2), 217–232.

